# Supplementary material for: Intraspecific Fine-Root Trait-Environment Relationships across Interior Douglas-Fir Forests of Western Canada
Source: Plants (Basel). 2019 Jun 30;8(7):199. doi: 10.3390/plants8070199 (PMC6681360; doi:10.3390/plants8070199)
Supplement: Supplementary file 1 [file plants-08-00199-s001.pdf]

Supplementary Materials

Figure S1 Ordination plot **(A)** and **(B)** associated scores of samples across a biogeographic gradient based on principal component analysis of fine root traits of second-order roots **(a)** and third-order roots **(b)** of interior Douglas-fir. C, root carbon concentration (%); N, root nitrogen concentration (%); SRA, specific root area (cm^2g^{-1}); SRL, specific root length (mg^{-1}); RTD, root tissue density ($mgcm^{-3}$); BrIntensity, branching intensity (cm^{-1}); DBI, dichotomus branching index. Branching intensity was not included in the ordination plot for third-order roots as it is calculated as the number of first- order root/ length of second- order root.

(A)

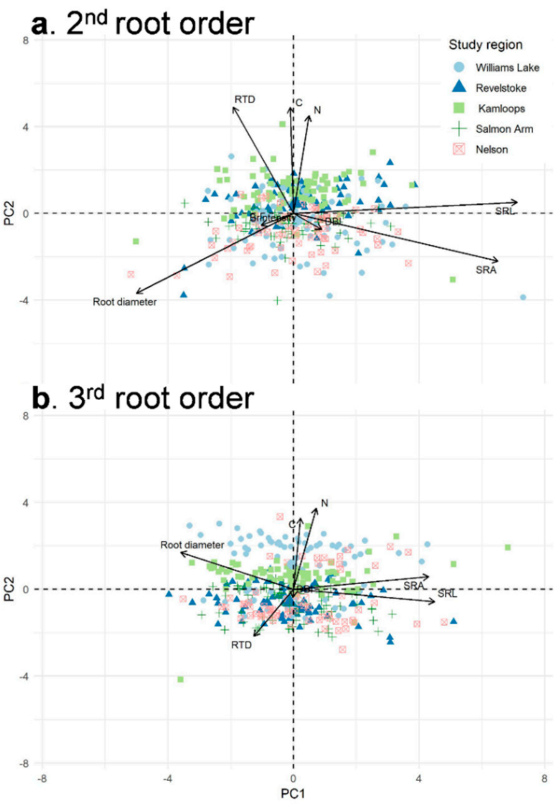

(B)

**1st order**	PC1 (30.33%)	PC2 (21.93%)	PC3 (15.10%)
SRL	**0.94**	0.22	-0.18
RTD	-0.60	0.32	**-0.66**
Root diameter	-0.44	-0.57	**0.64**
SRA	**0.95**	-0.01	0.18
DBI	0.15	-0.17	-0.11
Branching intensity	-0.06	-0.27	-0.21
Root N	-0.15	**0.76**	0.33
Root C	-0.17	**0.78**	0.37
**2nd order**	29.10%	20.58%	17.09%
SRL	**0.97**	0.07	-0.10
RTD	-0.26	**0.67**	-0.64
Root diameter	**-0.69**	-0.50	0.46
SRA	**0.89**	-0.30	0.24
DBI	0.12	-0.10	-0.22
Branching intensity	-0.14	-0.08	-0.15
Root N	0.06	**0.62**	0.58
Root C	-0.01	**0.67**	0.52
**3rd order**	35.95%	22.03%	17.77%
SRL	**0.97**	-0.12	0.07
RTD	-0.27	-0.47	**0.80**
Root diameter	**-0.78**	0.37	-0.43
SRA	**0.93**	0.12	-0.26
DBI	0.00	-0.10	-0.10
Branching intensity	*NA*	*NA*	*NA*
Root N	*0.16*	***0.81***	0.26
Root C	0.04	**0.71**	0.51

Figure S2 Distribution of branching intensity and dichotomous branching index values **(a)** across a biogeographic gradient and **(b)** variance partitioning of architectural traits at different hierarchically structured ecological scales (region, site, tree cluster and fine-root branch). WL, Williams Lake; R, Revelstoke; K, Kamloops; SA, Salmon Arm; N, Nelson. For (a), each data point represents one measurement for one root branch of interior Douglas-fir. *N* = 25 except for N2 where *N* = 15. The sign '+' within the boxes represents the mean value for comparison with the median value (centre line).

a.

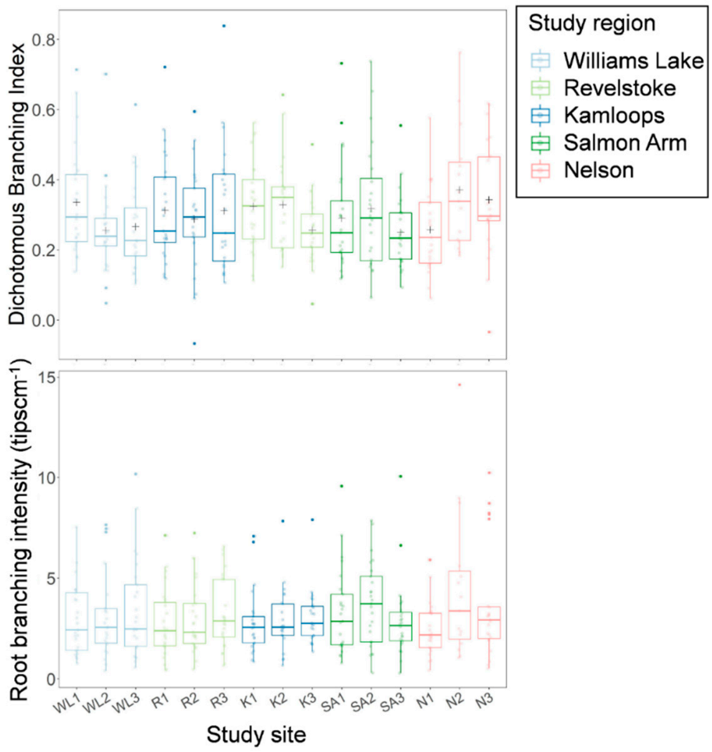

b.

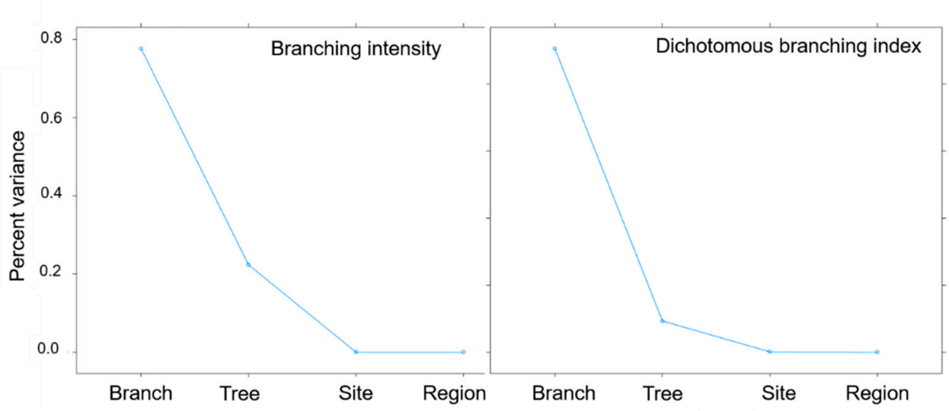

Figure S3 Ordination plot of study regions (five in total) across a biogeographic gradient based on principal component analysis of climatic, edaphic and site variables. MAP, mean annual precipitation (mm); MAT, mean annual temperature (°C); CN, soil carbon-to-nitrogen ratio; BA, Basal area (m^2ha^{-1}); CEC, effective cation exchange capacity ($cmol(+)kg^{-1}$); stand_comp, stand tree species composition. Principal component 1 explained 45.03 % and Principal component 2 explained 20.82 % of the data variation.

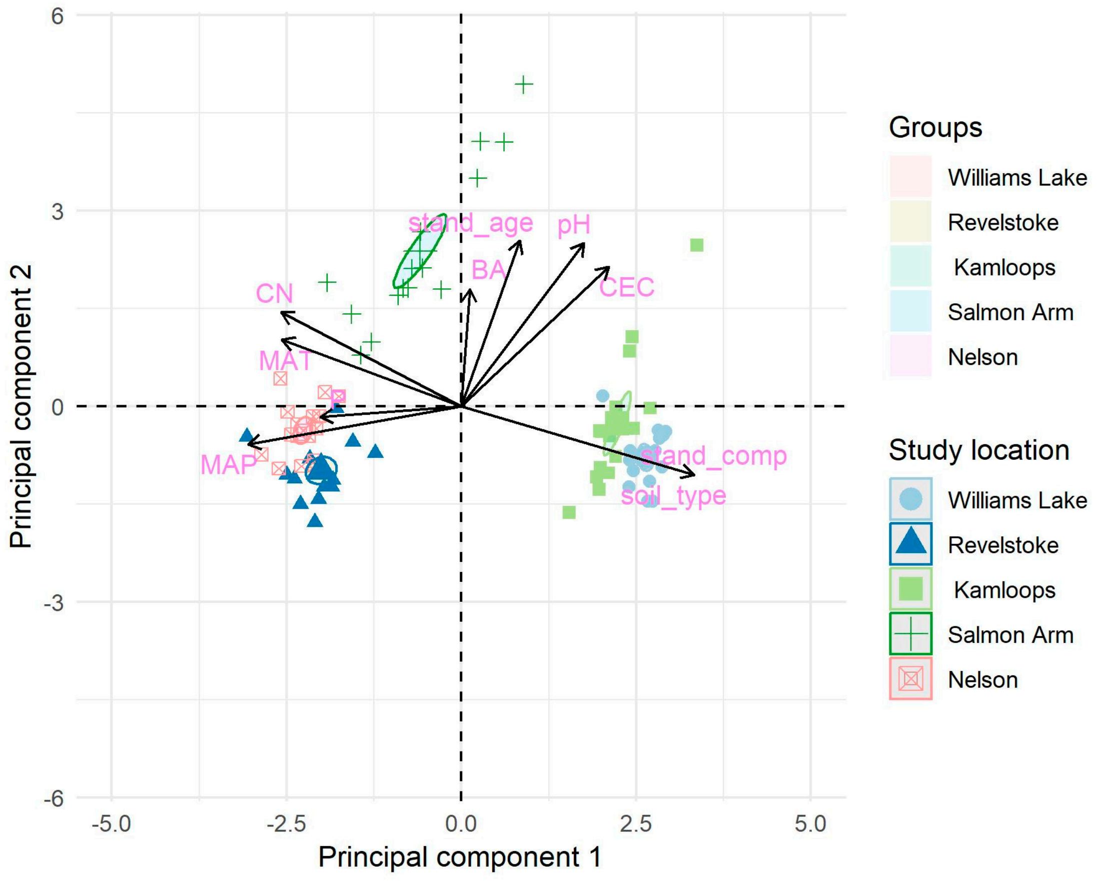

Figure S4 Description of morphological attributes of fine roots for three coniferous tree species encountered in this study. Description of root periderm texture and colour, root branching pattern and root tips habit are accompanied by exemplary pictures of root periderm and fine-root branching pattern. We validated the key with molecular genetic analysis of interior Douglas-fir (*Pseudotsuga menziesii* var *glauca*) and western hemlock (*Tsuga heterophylla* (Raf.) Sarg.; most similar morphologically). Samples were sent to the Appalachian laboratory at the Centre for Environmental Science (University of Maryland) and the findings of BLASTing Chloroplast DNA sequences from the rpl7 locus confirmed our expectations (accession numbers GQ999630.1, Douglas-fir and HQ846196.1, hemlock; Gugger et al., 2010). This figure was reproduced from Defrenne et al. (2019).

tree species	*Pseudotsuga menziesii* var. *glauca*<br>Interior Douglas-fir	*Tsuga heterophylla*<br>Western hemlock	*Thuja plicata*<br>Western redcedar
Periderm texture and colour	Older periderm parts form longitudinal, sinuous furrows of dark-grey colour.<br>Younger periderm is amber with some bright yellow strips .<br>Inner layers are bright orange to pale yellow.	Older periderm is amber with very fine longitudinal dark brown stripes. Brittle, detaches in chunks.<br>Old periderm on coarser roots is dark grey with reddish brown stripes.<br>Young periderm is smooth, dark red and forms a thin layer.<br>Inner layers are white.	Older periderm is brittle and scrapes off in slab. Forms fine furrows of amber colour.<br>Young periderm forms bright red fine strips, brittle and smooth. Inner layers are ochre to pale beige.
Branching pattern	Irregularly ramified branches, very tortuous.<br>No clear difference in size among fine root orders.	Regularly ramified and straight lined structured.<br>Tips are commonly grouped along root axis.<br>Tips are shorter and finer (and redder) than higher order roots.	Regularly ramified.<br>straight lined structured, no difference in size among fine root orders. Large branching angle.
Root tips	Forming ectomycorrhizas<br>Mostly thick, straight tips, pinnately.<br>tortuous tips with dichotomous branching.	Forming ectomycorrhizas<br>Mostly irregular branched and tips are bent.	Forming arbuscular mycorrhizas<br>long straight tips.

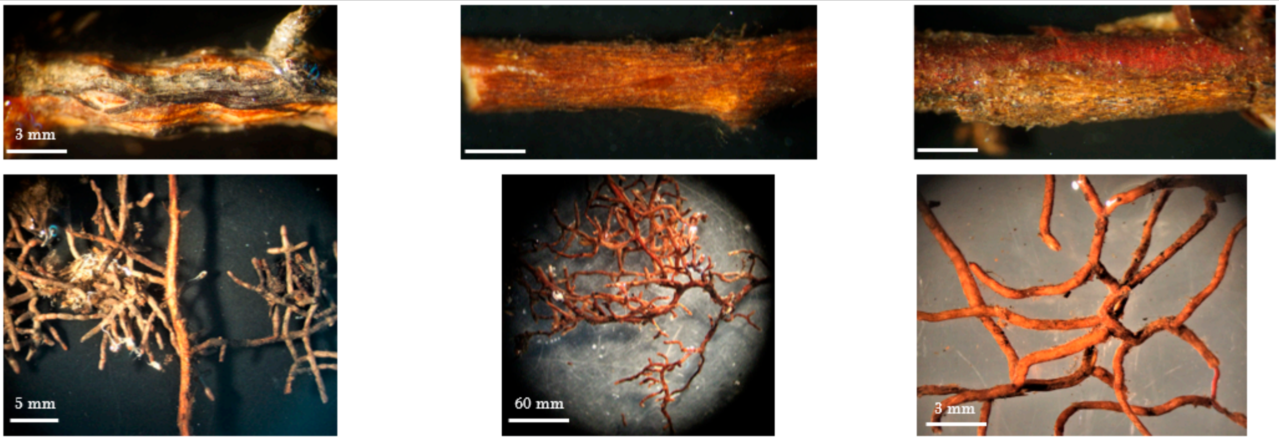

Table S1 Means and standard error (SE) of fine-root morphological, chemical and architectural traits of interior Douglas-fir across a biogeographic gradient made of five study locations

			Morphological root traits								Chemical root traits						Architectural root traits				
Study location	Root order	*N*	SRL (mg^{-1})		SRA (cm^2g^{-1})		RTD ($mgcm^{-3}$)		Root diameter (mm)		Root C %		Root N %		Root C:N		*N*	BrIntensity (cm^{-1})		DBI	
			Mean	SE	Mean	SE	Mean	SE	Mean	SE	Mean	SE	Mean	SE	Mean	SE		Mean	SE	Mean	SE
Williams Lake	1	75	16.97	0.43	213.60	4.21	470.50	10.10	0.41	0.01	40.35	0.25	1.02	0.02	40.43	0.69					
	2	75	10.51	0.37	172.20	4.05	449.00	11.33	0.54	0.01	44.55	0.29	1.10	0.02	41.15	0.49	75	3.15	0.24	0.27	0.01
	3	75	3.75	0.18	105.10	3.25	426.60	9.41	0.93	0.02	49.02	0.16	1.03	0.02	48.28	0.73					
Revelstoke	1	75	16.12	0.46	200.70	3.63	505.80	9.18	0.40	0.01	44.24	0.08	1.02	0.01	43.62	0.25					
	2	75	10.90	0.41	164.30	3.32	505.40	9.39	0.50	0.01	46.14	0.11	1.04	0.01	44.60	0.41	75	2.99	0.19	0.30	0.02
	3	75	4.00	0.20	99.00	2.68	503.70	8.27	0.84	0.02	48.34	0.12	0.81	0.01	60.08	0.53					
Kamloops	1	75	15.51	0.39	185.30	4.04	559.60	12.95	0.39	0.00	42.54	0.23	1.25	0.01	34.23	0.27					
	2	75	10.53	0.37	157.20	3.33	523.90	10.70	0.49	0.01	45.91	0.17	1.22	0.01	37.86	0.48	75	2.91	0.17	0.30	0.01
	3	75	4.07	0.22	99.71	3.60	494.50	13.44	0.86	0.02	49.82	0.10	0.94	0.01	54.16	0.91					
Salmon Arm	1	75	15.97	0.38	197.30	3.68	529.30	10.83	0.40	0.01	42.17	0.08	0.87	0.03	48.35	0.16					
	2	75	10.34	0.30	161.40	3.29	513.30	11.20	0.50	0.01	44.69	0.07	0.84	0.00	53.36	0.29	75	3.32	0.24	0.28	0.02
	3	75	4.06	0.19	101.30	2.72	493.00	9.58	0.84	0.02	48.00	0.10	0.80	0.01	60.28	0.52					
Nelson	1	65	16.88	0.57	203.00	4.51	513.30	11.33	0.38	0.01	39.51	0.53	0.84	0.03	48.89	0.94					
	2	65	10.91	0.43	165.50	3.74	495.50	9.08	0.51	0.01	43.93	0.16	0.81	0.01	54.14	0.38	65	3.39	0.33	0.32	0.02
	3	65	4.37	0.21	113.10	3.82	445.00	10.58	0.85	0.02	47.49	0.20	0.86	0.01	55.53	0.57					

N, sample size; SRL, Specific root length; SRA, Specific root area; RTD, Root tissues density; Root C:N, Root carbon-to-nitrogen ratio; BrIntensity, Branching intensity (calculated as the number of first- order root/ length of second- order root); DBI, Dichotomous branching index, values closer to 0 indicate a dichotomous branching pattern and values closer to 1, a herringbone branching pattern, see Beidler et al. (2015).

Table S2 Spearman's correlation coefficient for pairwise root order (first three root orders) relationships

1st order	SRL	RTD	Root diameter	SRA	DBI	Branching intensity	Root N
RTD	**-0.26**						
Root diameter	**-0.67**	**-0.46**					
SRA	**0.83**	**-0.73**	**-0.20**				
DBI	0.05	-0.08	0.00	0.09			
Branching intensity	0.09	0.04	-0.11	0.04	-0.03		
Root N	-0.01	0.08	-0.06	-0.04	-0.04	0.01	
Root C	-0.02	0.07	-0.05	-0.03	-0.09	0.01	**0.40**
2nd order							
RTD	-0.07						
Root diameter	**-0.81**	**-0.46**					
SRA	**0.86**	**-0.51**	**-0.44**				
DBI	0.07	-0.01	-0.06	0.07			
Branching intensity	0.10	-0.04	-0.10	0.10	-0.03		
Root N	0.02	0.00	-0.01	0.02	-0.02	0.00	
Root C	0.02	0.06	-0.09	-0.02	-0.06	0.03	**0.44**
3rd order							
RTD	**-0.17**						
Root diameter	**-0.88**	**-0.23**					
SRA	**0.92**	**-0.48**	**-0.67**				
DBI	-0.03	0.02	0.02	-0.03			
Branching intensity	NA	NA	NA	NA	NA	NA	
Root N	0.10	-0.27	0	**0.17**	0.00	NA	
Root C	0.01	-0.01	0	0.02	-0.02	NA	**0.46**

SRL, Specific root length; RTD, Root tissues density; SRA, specific root area; DBI, Dichotomous branching index, values closer to 0 indicate a dichotomous branching pattern and values closer to 1, a herringbone branching pattern, see Beidler et al. (2015); Branching intensity (the number of first- order root/ length of second- order root), this was not assessed for third-order roots; Root C, Root carbon concentration (%); Root N, Root nitrogen concentration (%). Bold values indicate statistically significant correlation at $P<0.05$. NA, not applicable.

Table S3. Stand properties of the 15 study sites selected across a biogeographic gradient in Western Canada.

Study region	Stand within region	Stand properties					
		Dominant tree species	Basal area m^2ha^{-1} (% Douglas-fir)	Stand Age (years)	Soil type (texture)	Humus type	rooting depth (cm)
Williams Lake	WL1	*Pseudotsuga menziesii* *Pinus contorta*	22.1 (98%)	125	luvisol (C)	mor	58
	WL2		15.3 (96%)	141	luvisol (SiCL)	mull	34
	WL3		18.5 (97%)	106	luvisol (SiCL)	mull	37
Revelstoke	R1	*Pseudotsuga menziesii* *Tsuga heterophylla* *Thuja plicata*	60.6 (66%)	106	brunisol (SiCL)	mor	45
	R2		38.4 (66%)	83	brunisol (L)	moder	50
	R3		47.9 (72%)	104	brunisol (L)	moder	50
Kamloops	K1	*Pseudotsuga menziesii*	56.4 (100%)	126	luvisol (SiC)	moder	40
	K2		37.6 (100%)	94	luvisol (L)	moder	40
	K3		48.3 (100%)	104	luvisol (L)	moder	30
Salmon Arm	SA1	*Pseudotsuga menziesii*	98.5 (50%)	142.5	brunisol (SiL)	mull	80
	SA2	*Larix occidentalis*	81.8 (58%)	140	brunisol (L)	mor	76
	SA3	*Tsuga heterophylla* *Thuja plicata*	80.6 (39%)	147	brunisol (L)	mull	40
Nelson	N1	*Pseudotsuga menziesii*	49.8 (65%)	115	brunisol (SL)	mor	60
	N2	*Larix occidentalis*	65.4 (29%)	100	brunisol (SL)	moder	37
	N3	*Tsuga heterophylla* *Thuja plicata*	56.0 (55%)	104	brunisol (SL)	moder	49

The soil texture was assessed on the first B horizon. L, loam; SiC, silty clay; SiCL, silty clay loam; SiL, silt loam; SL, sandy loam. This table was reproduced from Defrenne et al. (2019).

References

Beidler, K.V.; Taylor, B.N.; Strand, A.E.; Cooper, E.R.; Schönholz, M.; Pritchard, S.G. Changes in root architecture under elevated concentrations of CO 2 and nitrogen reflect alternate soil exploration strategies. New Phytologist 2015, 205, 1153–1163.

Defrenne, C.E.; Philpott, T.J.; Guichon, S.H.A.; Roach, W.J.; Pickles, B.J.; Simard, S.W. Shifts in ectomycorrhizal fungal communities and exploration types relate to the environment and fine-root traits across interior Douglas-fir forests of western Canada. Front. Plant Sci. 2019, 10.

Gugger, P.F.; Sugita, S.; Cavender-Bares, J. Phylogeography of Douglas-fir based on mitochondrial and chloroplast DNA sequences: testing hypotheses from the fossil record. Molecular Ecology 2010, 19, 1877–1897.
